# This cookie will save the planet! The effect of a private sustainability claim on consumers’ expectations

**DOI:** 10.1016/j.heliyon.2023.e14206

**Published:** 2023-03-05

**Authors:** Gerarda Caso, Emanuele Blasi, Luigi Cembalo, Riccardo Vecchio

**Affiliations:** a Department of Agricultural Sciences, University of Naples Federico II, Via Università, 100, Portici, NA, 80055, Italy; b Department for Innovation in Biological, Agro-Food and Forest Systems, University of Tuscia, Via San Camillo de Lellis snc, Viterbo, VT, 01100, Italy

**Keywords:** BDM mechanism, Food shopping responsible, Sustainable agriculture, Claim, Halo effect

## Abstract

Current study investigates whether a private, sustainability claim has an impact on individuals' sensory and non-sensory expectations (halo effect) and quantifies such impact on individual monetary preferences. An incentive-compatible artefactual field experiment was performed by recruiting regular buyers and consumers of the investigated product (cookies). Results reveal that the sustainable agriculture claim generates high (unrelated) expectations and a statistically significant premium price compared to the conventional counterpart. Additionally, these expectations, together with respondents’ trust in the claim, are the drivers of the price premium. Findings suggest the need for policy makers and consumer advocates to scrutinize the potential drawbacks of private sustainability claims on food products.

## 1 Introduction

A halo effect occurs when a specific signal influences the overall evaluations of a product, which subsequently biases the evaluations of other attributes [1,2]. Labelling, in this sense, represents the specific signal that captures consumer's attention and influences the product overall evaluation, allowing to discriminate between alternatives during the purchase [3,4]. The communicative performance of labelling is closely dependent on the correct interpretation of the message by the end user nevertheless, in practice, this evaluation is not always in the right direction. Research proves that when information is not directly available, it is inferred from positively or negatively related context cues [5]. For example, the calorie content of a food is unjustifiably underestimated if the product is labelled “gluten-free” [6] or “low-carbohydrate” [7]. If, in addition to subjective interpretations of the label - despite dealing with quantifiable aspects (as in the case of calorie content) - belief attributes (*i.e.*, not directly verifiable by the consumer either before or after purchase) are carried on the packaging, a bias could further skew the final product evaluation. This is the case with foods bearing labels that imply sustainable features. Research has shown that product labelling related to purely sustainable aspects (*e.g*.: fair trade [8]), or fictitious [9], is often misunderstood or misinterpreted [[10], [11], [12]]. Indeed, consumers may mistakenly perceive a food product carrying a sustainable claim as a good with a superior sensory quality compared to a product without the claim. For example, just calling a product “eco-friendly” is enough to make consumers believe it tastes better than an objectively identical alternative [13]. Additionally, individuals might assign positive features to product attributes that are not linked to the claim. For example, “fair-trade” chocolate can be perceived as having a low-calorie content, despite the claim does not mention the nutrient content [14]. Such cognitive positivity bias – generally defines as halo effect - occurs when individuals use their overall positive evaluation of an attribute to make positive inferences about product's other attributes, without necessarily being aware of the origin of these inferences [1,2]. These inferences are the result of psychological processes that take place before consumption [15], when individuals integrate previously experienced (and stored) information with any newly present signal about the food, such as claims, until creating powerful mental expectations about what they will experience [16]. Expectations can enhance or degrade the perception of a product and thus influence purchase intention. Accordingly, labels with a positive feature such as a sustainable claim could enhance consumer perceptions and hedonic evaluations [16], as well as monetary preferences, and this is especially true for pro-environmental consumers [11,17].

Most of the available literature on the halo effect originating from sustainable claims has focused on “organic” food products [11,[18], [19], [20], [21], [22]]. For example, in the study by Lee and colleagues [23], participants rated for each pair of products presented (cookies, chips, and yogurt) those with the organic label as nutritionally better than the counterpart without claims, despite being identical. Similarly, the results of Prada et al. [11] showed that organically produced foods were perceived as healthier, tastier, and less caloric than conventionally produced foods. Indeed, organic food have a strong health connotation [19], albeit scientific evidence for these benefits seems limited (for a complete overview, see Ref. [24]). Therefore, the halo effect generally associates multiple health-related benefits to organic foods, such as lower calorie content [14,21], superior nutritional value [23], better taste [21,22] and evoking positive emotions [21]. Nevertheless, organic certification was exclusively developed to inform about the applied production methods.

Most of the analyses performed by scholars to support the halo effect have concentrated on hedonic liking evaluations of foods with/without specific claims. Participants in the study by Loz et al. [25] reported that coffee and chocolate tasted significantly better when labelled as fair trade compared to the conventional counterparts. Similarly, in Sörqvist and colleagues’ study [17], participants reported that they preferred the taste of eco-friendly coffee over the other cup, although (in reality) the two cups contained identical coffee: associating the sustainable product with a more favorable perceptual experience. Further research has proved that bananas, grapes and sultanas taste better when labelled eco-friendly [13]; and wine tastes more intense, pleasant, and fruity when labelled organic [26,27].

Moreover, a relevant aspect that could be related to the halo effect is individuals’ willingness to pay generated by the specific claim carried by the food. Comparing the same products with and without sustainability claims, evidence suggests that consumers are willing to pay a premium price for foods carrying a claim [18,21,[28], [29], [30], [31]], with significant differences in relation to product type, specific sustainability claim and cultural background of the respondent.

The recent meta-analysis of Li and Kallas [32] well depicts the global phenomenon of high consumer interest towards sustainable food products. Nevertheless, the overwhelming majority of available studies analyze individuals' preferences for sustainability labelled foods compared to conventional products and explore preferences' core drivers [[33], [34]]. The current study focuses on the effect of a completely new private sustainable claim - not launched in the market at the time of the experiment and framed as “sustainable agriculture” - on consumers’ expectations, extending recent research which has mostly investigated organic, fair-trade, or eco-friendly food label [[10], [11], [12]]. Specifically, the study explores how this claim developed by a market-leading food company and added to an existing cookies package (already on the supermarket shelves) is perceived and interpreted by consumers and whether it generates unjustified sensory and non-sensory expectations (halo effect) compared to the conventional counterpart. Furthermore, individual monetary preferences are analyzed to verify if the sustainable claimed food receives a price premium and what factors drive this possible premium.

An incentive-compatible artefactual field experiment was performed by recruiting regular buyers and consumers of the investigated product [35,36], to test the following research hypotheses.

1) The interpretation of the private, sustainable agriculture claim generates a halo effect by promoting unjustified sensory and non-sensory expectations.
2) Individuals are willing to pay a premium price for the product with a private, sustainable agriculture claim.
3) The halo effect is a core driver of individuals' monetary preferences.

Current research provides a significant contribution to the literature by revealing new insights into consumer expectations arising from a private, sustainable claim and shedding light on the impact of the halo effect on individual monetary preferences. The study adds to current knowledge collecting both sensory and non-sensory evaluations for a real-world product (maximizing realism) and detecting expectations’ impact on non-hypothetical consumer monetary preferences. Additionally, evaluating the potential impact of a non-fictitious private claim soon to be launched in the market, it powerfully informs the debate on how this type of claims can potentially mislead consumers.

## 2 Material and methods

### 2.1 Sample selection

The experiment was conducted between November and December 2019 in the Experimental Economics and Consumer Science Laboratory of the University of Naples. The experiment involved a random, convenience sample of 150 respondents, all responsible of household food shopping, regular consumers of breakfast cookies and buyers, aged over 18. The sample was recruited through phone messages and word-of-mouth. The content of the message informed of the exclusively scientific purpose of the experiment, the approximate length of the session (45 min) and the broad topic of the study (food purchases). The message also provided a calendar of experimental sessions to facilitate the choice of participants in accordance with their respective needs. Fifteen experimental sessions were organized with ten individuals each. Table 1 shows the socio-demographic characteristics of the interviewees, aged between 19 and 78 years.

**Table 1:** Sample characteristics (N = 150).

*Sex at birth*	Women	68.0%
	Men	32.0%
***Age***	Mean 40.65 S.D. 14.23	
***Household size***	1 person	4.7%
	2 persons	11.3%
	3 persons	27.3%
	4 persons	36.7%
	>5 persons	20.0%
***Children in the household***	Yes	33.0%
	No	67.0%
***Education***	Elementary School	0.7%
	Secondary school	8.6%
	High school diploma	48.0%
	Bachelors degree	36.0%
	Master/PhD	6.7%
***Monthly income***	Low	55.3%
	Medium	43.4%
	High	1.3%
***Occupation***	Entrepreneur	1.4%
	Employee	24.7%
	Freelancer	11.3%
	Housewife	19.3%
	Retiree	6.0%
	Teacher	5.3%
	Unemployed	32.0%

The sample average age is 40.65 years (S.D. 14.23), with a larger share of women (68%) than men. The mean household size is 3.61 and 33% of the sample have children under 12 years old. 57.3% of respondents hold an education equal to or less than a diploma, and 98.7% states to have a medium-low range of household monthly income. Concerning the occupation, the most represented category is unemployed (32%), followed by employees (public or private) (24.7%), housewives (19.3%), and freelancers (11.3%).

The following sections provide a complete description of the entire experimental procedure, the stimuli applied, the on-line questionnaire and how the collected data were analyzed. All data were collected, recorded and managed in accordance with the Declaration of Helsinki, and with the “Italian Personal Data Protection Code” (Law Decree no. 196 of June 30, 2003) as certified by ethical waiver declaration provided by the chief of the Department.

### 2.2 Experimental procedure

The experiment followed a within-subject design: all participants evaluated two cookie packages with the same type of information and without tasting the products. Products used in the experiment were two packs of cookies: a conventional one and one with the sustainability claim (product details are shown in section 2.3). At the time of the experiment the new package and related sustainability initiative were not released to the public by the brand. Thus, participants in the experiment could not know the specific characteristics of the initiative neither were previously stimulated by specific narratives on the product new features.

To investigate the monetary preferences of individuals for the products analyzed^1^, the incentive compatible experimental elicitation technique used was the Becker-DeGroot-Marschak mechanism (BDM; [37]). BDM is a widely applied mechanism in food marketing (see, among others, [38,39]) to determine value in non-hypothetical contexts [40]. The selection of BDM in the current study was related to two of its core features: the mechanism comprehension is quite high among participants [41,42] and it avoids experimenters to recruit exactly the same number of individuals for each experimental session [43]. In a full-bidding BDM, each participant makes a bid equal to their maximum willingness-to-pay (WTP) for all the products being sold. These bids are then compared with a randomly drawn price from a uniform distribution. If the participant bid is equal or greater than the randomly drawn price, the participant purchases the product at the drawn price; conversely, all those who bid below the extracted price will not buy the product [44]. The optimal strategy for all rational participants is to offer exactly their maximum WTP as both excessively high and low bids are equally penalized [44,45].

**Fig. 1:**
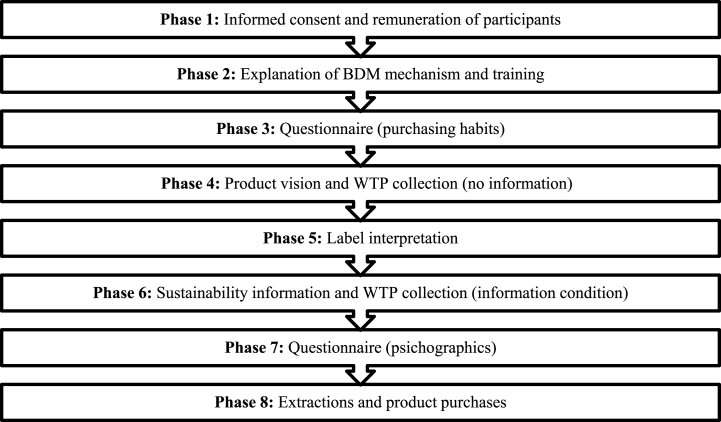
Phases of the experiment.

The complete experimental procedure included eight phases (see Fig. 1); hereafter described in detail.

**Fig. 2:**
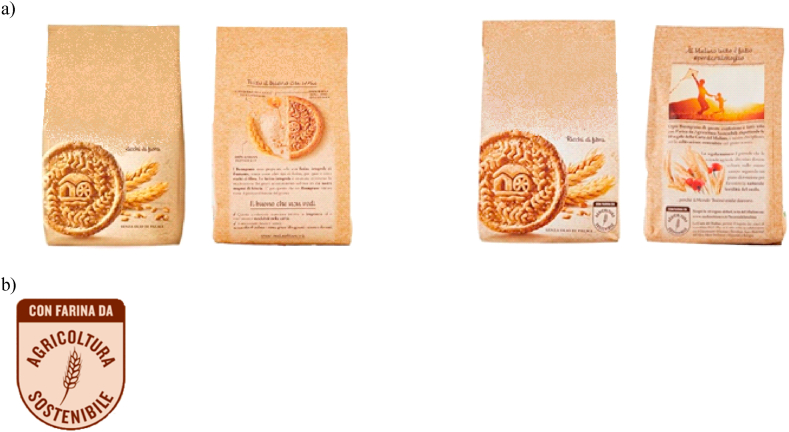
Front and back of the two packages of cookies: on the left the conventional pack, on the right the sustainable pack with the claim (**2a**); Detail of sustainability claim, - with flour from sustainable agriculture - literal translation of the text (**2b**).

In the first phase, participants were welcomed into the laboratory and were invited to occupy an available individual-post. Each post was provided with a personal computer, a unique identification code (able to guarantee anonymity throughout the experiment) and with paper informed consent form for data processing. Participants were invited to complete the informed consent form. At the same time as the withdrawal of the form, the participation fee of €10 was distributed to each interviewee to reward the time spent in the laboratory. The interviewees were asked not to communicate with each other for the duration of the experiment and to express their free opinions. In the second phase, the general procedure of the entire experiment was fully explained. To familiarize participants with the BDM mechanism, participants were trained through simulations. In this specific case, a chocolate bar was used in two of its variants. Once we made sure everyone understood the mechanism, we proceeded to the next phases. In the third phase, a short-computerized questionnaire was administered to the interviewees to collect food purchasing and consumption habits. Then, the real BDM mechanism was carried out in two WTP elicitation rounds: the two packages of cookies (the conventional one and the sustainable one, Fig. 2) were shown to the participants together with a visual aid (images projected on slides and individual pc monitor). In the first round (fourth phase), participants had no information about products except visual assessment (condition without information). Participants were asked to submit their maximum WTP for each cookie pack presented. In the second round (sixth phase), a general description of sustainability claim was disclosed (information condition). At the end of the description, the participants were invited to express their maximum WTP for the same pack of cookies again. Between the two rounds of WTP elicitation, particularly before providing the additional information about ecosystem services [46] and the label itself, interviewees provided their personal understanding of the claim from a list of likely interpretations (fifth round). In the seventh phase the participants were asked to complete the questionnaire (more details refer to section 2.4). The session ended with the purchase of the products draw according to the BDM mechanism. No deceptive practices were applied in the experiment. Complete experimental instructions and session details are available from the authors upon request.

### 2.3 Stimuli

Two commercial packages of cookies were used in the experiment (Fig. 2a), one with the private claim of sustainable agriculture and a conventional counterpart. The two front packages had the same, identical characteristics (package color, shape, weight, brand). The only difference between the packages was the presence of the sustainable agriculture claim on one front package at the bottom left. Whilst the back of the packages was different: the conventional package showed the classic recipe and ingredients information, the cookie package with the claim showed information related to sustainable agriculture (Fig. 2b). As described above, cookie packs were shown to respondents both directly (*i.e.*, handing out the packages during the experiment) and through visual aids. In the “no information” condition, respondents declared their maximum WTP for each package just by observing the products. In the second phase (information condition), to capture the effect generated by the sustainable claim, a general description of ecosystem services [46] was provided (via individual pc) which emphasized how “cookies produced with flour from sustainable agriculture safeguard ecosystems by promoting the natural fertility of the soil”. Then respondents were asked to express their individual WTP for the two packages.

### 2.4 Questionnaire design and applied measurements

The pc-based questionnaire was organized into six sections (Table 2 provides a general overview of the design and applied measurements). The first section gathered information about cookie consumption habits: respondents were asked to state purchasing frequency and the average price paid for the last package of cookies purchased. The second section measured the level of understanding of sustainable claim shown on cookie package. Respondents were asked to choose the correct meaning of the claim from a list of sixteen potential responses [47]; participants could select more than one answer within this list. The third section assessed individual expectations, participants stated their overall evaluation for both cookie packages for seven selected attributes [48]. This multi-attribute scale included the following statements: “I expect the quality/particularity/attractiveness/taste/nutritional properties/safety/health of this product, compared to other similar products, is” followed by a differential 7-point semantics scale, which varied from 1 (much worse) to 7 (much better) [48]. In the fourth section, trust in sustainability certification was measured using a three-item scale that was originally developed by Hess and Story [49]. Respondents rated the degree of agreement/disagreement for each statement on a seven-point scale (1 = “totally disagree” and 7 = “totally agree”). The fifth section assessed concern for food sustainability; measured by fourteen different sustainability statements related to the impacts of the food sector, selected from the scale proposed by Grunert et al. [47] and previously used in other research on sustainable food consumption [31]. For each item, participants expressed their level of concern on a seven-point scale (ranging from 1 = “slightly worried” to 7 = “extremely worried”). In the sixth section, socio-demographic variables were collected (*i.e.*: sex at birth, age, education, monthly income, number of household members, presence of children and occupation).

**Table 2:** Questionnaire design and applied measurements.

Questionnaire Section	Topic	Applied measurement	Example
I	Cookie consumption habits	Multiple-choice [our elaboration]	*How much did you pay for the last package of cookies you bought?*
II	Label understanding	Multiple-choice [47]	*Minimising (soil) contamination when producing food*
III	Product expectations	Range: from 1 (= much worse) to 7 (= much better) [48]	*I expect the quality of this product, compared to other similar products, is..*
IV	Trust in sustainability certification scale	Range: from 1 (= totally disagree) to 7 (= totally agree) [49]	*The promises of product sustainability certifications are probably true*
V	Concern for food sustainability scale	Range: 1 (= slightly worried) to 7 (= extremely worried) [47]	*Deforestation of the rain forest*
VI	Socio-demographic variables	Multiple-choice/open-ended [our elaboration]	*Number of family members*

### 2.5 Econometric analysis

As stated in the introduction, the current research addresses three research hypotheses, that are: evaluating if a “sustainable agriculture” claim has a halo effect, quantifying such impact on individuals' WTP and verify if the halo effect is a core driver of individuals’ WTP.

Paired-sample *t*-tests investigated whether the expectations (relative to several product characteristics) generated by the cookie package carrying the claim were significantly different from the expectations generated by the conventional counterpart. Since the sustainable agriculture label is the only difference between the two packages, significant deltas (sustainable minus conventional) underscore the positive halo effect generated. To answer the second research question, paired-sample *t*-test and Wilcoxon signed-rank tests compared respondents' self-reported WTP. It is worth clarifying that our interest is on the difference in WTP between the sustainable and the conventional products (in the two rounds) rather than the absolute WTP for each package of cookies. Since the experimental procedure was designed as a within-subjects, a seemingly unrelated regression (SUR) model was implemented. The SUR is a multivariate linear regression model suited to contexts where the estimation of a system of equations is needed [50]. It allows efficient estimates, compared to least square single equation estimations, by weighting based on the covariance of the residuals of the individual regressions. To illustrate, the SUR applied in the current paper consist of two linear regression equations — one for each delta WTP (sustainable minus conventional in round 1 and sustainable minus conventional in round 2), in which the error terms are assumed to be independent across individuals and correlated across equations. More formally, the following system of equations was estimated for the *i*-th respondent:

(1) $$\left\{ \begin{array}{c} {\Delta WTP}_{R1,i}\\ {\Delta WTP}_{R2,i} \end{array}\right.\;\begin{array}{c} \;=x^{\prime }\beta_{R1}+e_{R1,i}\\ \;=x^{\prime }\beta_{R2}+e_{R2,i} \end{array}$$

where the two dependent variables *ΔWTP* represent the differences in the WTP between the sustainable cookie package and the conventional cookie package in the two respective rounds; *x* is a vector of explanatory variables (Table 3 lists and describes the explanatory variables); and *e* are the error terms. In each equation, the estimate of statistically significant coefficients *β* identifies and measures the corresponding determinants of individual WTP. The potential significance of ΔExpectations variable suggests that the halo effect actually drives respondents' price premium for the package holding the claim. Specifically, two SUR models were estimated: one without participants characteristics (Model 1) and one that includes individual-related variables (Model 2). The collected data were processed using the STATA 16 statistical software.

**Table 3:** Summary of variables applied in the SUR models.

Variable	Description	Scale and variable type	Mean (S.D.)
Trust	Trust in eco-labelling	Continuous	4.12 (1.07)
ΔExpectations	Expectation of product attributes [ΣΔ = Σ (S–C)]	Ordinal: 7 (C > S) to 7 (S > C)	0.25 (0.72)
Understanding	Correct interpretation of the sustainable claim	Dummy: 1 = all correct, 0 = otherwise	0.47
Productive process concern	First factor of the sustainability concern scale	Continuous	
Environmental concern	Second factor of the sustainability concern scale	Continuous	
Ethical concern	Third factor of the sustainability concern scale	Continuous	
Sex at birth	Respondent sex at birth	Dummy: 1 = Women, 0 = men	0.68
Age	Respondent age in years	Continuous	40.65 (14.23)
Household size	Number of household members	Ordinal: 1 (= one person) to 5 (= more than five persons)	3.61 (1.16)
Children	Presence of children (<12 years) living in the household	Dummy: 1 = present, 0 = not present	0.36
Education	Highest educational level acquired	Dummy: 1 = graduate or more; 0 = not graduated	0.43
Monthly income	Household monthly income compared to national average	Dummy: 1 = higher, 0 = equal or lower	0.45
Occupation	Respondent occupation	Dummy: 1 = worker, 0 = unemployed	0.68
Cookie shopping frequency	Cookies purchasing frequency	Ordinal: 1 (= seldom) to 5 (= very often)	3.50 (0.98)
Average price paid for a cookie package	Price of last cookie package purchased compared to average market price (€2)^1^	Dummy: 1 = higher, 0 = equal or lower	0.34

## 3 Results

### 3.1 Participants’ characteristics

The final sample includes 150 individuals living in Naples province (Southern Italy). These are individuals responsible for household food purchases, which often (41.3%) or very often (14.7%) purchase cookies for breakfast. Most respondents (46%) paid between 1.50€ and 2.00€ for the last package of cookies purchased, followed by 34% who instead paid more than 2.00€, while 20% paid less than 1.50€. To explore respondents sustainability interest and concern, two psychographic scales assessed individual trust in the sustainable certification on food products [49] and concern for sustainability in general [47]. Table A1 in the appendix provides further details of scale items and scores. The calculated values of Cronbach's alpha (α) for these scales were above 0.70, thus indicating a high degree of internal consistency. From the three items related to the trust in sustainable certifications scale (α = 0.77) the interviewees, on average, state confidence in the sustainability certifications present on the product packaging, as a real commitment to environmental protection (*M* = 4.12). Similarly, the fourteen items that measure concern about different aspects of sustainability related to food production (α = 0.88) reveal quite high average values (ranging between 4.63 and 6.36). An exploratory factor analysis of these 14 items of the sustainability concern scale by Grunert et al. [47] generated 3 factors (see Table A2 in Appendix for further details). The first factor concerns the damage caused by the production process in terms of emissions and quantity of energy/materials used; the second dimension is associated with environmental damage caused by the inappropriate human use of natural resources; the third factor evokes the ethical aspects relating to poor treatment conditions for both workers and animals. These factors will be hereafter called as “Production process concern”, “Environmental concern” and “Ethical concern” respectively.

### 3.2 Halo effect of the sustainable claim: expectations and understanding

To answer the first research question, the descriptive statistics frame a first perceptual representation of the different expectations raised by the sustainable product (Table 4). The expectations for the product in general, both conventional and sustainable, are positive and all above the average value (>5). Paired samples *t*-tests confirm that the mean values for six out of seven expectations are statistically significant and higher for the sustainable cookies compared to the conventional counterpart. Since the sustainable agriculture claim is the only difference between the two packages of cookies, we can therefore accept the hypothesis that the difference between the reported expectations is related to the label itself and thus to the positive halo effect it generates. It seems, therefore, possible to state that the sustainable certification leads consumers to perceive the food product as of better quality/more particularity/more attractive/tastier/safer/healthier, highlighting a link between the sustainable process and product characteristics that has no concrete support.

**Table 4:** Expectations’ mean ratings (Standard Deviation in parenthesis) and absolute differences between sustainable-conventional (Delta).

Expectations	*Sustainable cookie*	*Conventional cookie*	*Delta*	*P-value*
Quality	5.80 (1.10)	5.47 (1.19)	0.33 (1.06)	<0.001
Particularity	5.54 (1.14)	5.20 (1.16)	0.34 (1.10)	<0.001
Attractiveness	5.35 (1.13)	5.09 (1.14)	0.26 (1.09)	0.004
Taste	5.57 (1.32)	5.37 (1.33)	0.20 (1.01)	0.016
Nutritional properties	5.77 (1.22)	5.68 (1.21)	0.09 (0.99)	0.288
Safety	5.85 (1.20)	5.63 (1.37)	0.22 (1.23)	0.030
Health properties	5.85 (1.27)	5.57 (1.36)	0.29 (0.95)	<0.001

To investigate the understanding of the message carried by the sustainable claim on the packaging, respondents could select from a list of sixteen specifications one or more options corresponding to their interpretation of the claim. The sixteen statements were about different aspects of sustainability and were discriminated as correct or incorrect if the specific sustainable claim applied in our study carried this detailed feature or not. In particular, only six statements actually conveyed the actual features of the case-study label. The results show that, although the six true specifications were the most selected (see Table A3 in the Appendix), there is great variety in the responses provided.

In addition to supporting farming communities and providing nature space in agro-ecosystems in favor of biodiversity, respondents associate the sustainable agriculture claim with completely unrelated aspects concerning packaging quantity and recycling, pricing policies and product distribution. This result underlines a new halo effect: the general positive sentiment towards the sustainable claim creates inferences about both objective characteristics and peculiarities of the product, without necessarily being aware of the origin and correlation of these inferences. To use this information efficiently, the list of 16 specifications was recoded into a single dummy: 1 if the respondent interpreted the claim correctly (*i.e.:* individuals who selected all six correct statements, and respondents who, by selecting a smaller number of responses – from one to five – inserted only sustainable aspects effectively carried by the claim), 0 in all other cases (*e.g.,* individuals who selected one or more correct interpretations but simultaneously at least one incorrect feature). In this manner a direct measure of the (potential) misleading effect generated by the claim was obtained.

### 3.3 Individual monetary preferences

To address the second research question and assess whether the inclusion of the sustainable agriculture claim provides a premium price to the cookie package, pairwise comparisons were conducted. In round 1, without providing additional information beyond the package view, the average bid for the sustainable cookie was €1.87; while the average bid for the conventional one was €1.81. Based on the paired-sample *t*-test, the two offers are statistically different (*p* < 0.001): sustainable package receives a €0.06 premium price (Table 5). Similarly, in Round 2, the average bid for the sustainable alternative was €1.99 statistically different and higher than the average bid for the conventional pack (€1.88). Thus, the sustainable product received on average a higher WTP than the conventional in both rounds. In particular, the transition from no information (Round 1) to additional information relating to the sustainable claim (Round 2), has increased the offers for the product of 0.11€: the information justifies a statistically higher price delta (*p* < 0.001). In addition, in according with Wilcoxon signed-rank test [51], the distributions for each round are statistically different between sustainable and conventional packages.

**Table 5:** Mean WTP for the two cookie packages (€) and statistically significance of differences.

Product	Mean	Median	Min.	Max	Delta (*t*-test)	P-value	Wilcoxon signed-rank test	P-value
*Round 1 – No Information*								
Sustainable	1.87 (0.69)	1.80	0.40	4.50	0.06 (0.20)	<0.001	ΔS1–C1	0.003
Conventional	1.81 (0.64)	1.70	0.40	4.00				
*Round 2 – Information*								
Sustainable	1.99 (0.75)	1.90	0.50	5.00	0.11 (0.28)	<0.001	ΔS2–C2	<0.001
Conventional	1.88 (0.72)	1.80	0	5.00				

Notes: The Wilcoxon test results are in line with other statistical tests. The mean WTP for the conventional cookie package also increases between round 1 and 2. This could be related to several issues; among these we highlight the possible influence of bounded rationality and experimental fatigue.

### 3.4 Drivers of individuals’ monetary preferences

To answer the third research question and test whether the halo effect generated by the claim justifies a higher WTP for the product with a sustainability claim, the SUR model was applied. The dependent variable is the delta, constructed as the difference between the WTP for the sustainable and conventional product in each round. The independent variables used in the models are described in Table 3. Preliminary analyses tested the correlation between the independent variables to ensure that there were no multicollinearity issues. Table 6 reports the estimated coefficients of the regression model without participants characteristics (Model 1) and including individual-related variables (Model 2). These SUR models reveal the drivers of premium price under uninformed and informed conditions. Due to space constraints hereafter, we discuss exclusively results of Model 1. In Round 1, a positive (albeit minimal) delta WTP was detected. The first equation in Table 4 shows how this delta is mediated by the confidence that respondents place in sustainable agriculture (the only discriminating factor between the two packs of cookies) and expectations. Indeed, as the level of trust placed in certification increases, the delta increases by € 0.011. Likewise, as the level of expectations increases, the delta surges by € 0.087. All other variables are not significant at the 10% level. In Round 2, the additional factor was the information on the sustainable agriculture practices used. Similarly in this case the only significant and positive variables are trust and expectations, with the impact of expectations on the ΔWTP equal to € 0.21. Another significant variable with a negative effect is the aspect of sustainability linked to the ethical dimension: a greater concern for this type of sustainability reduces the WTP for the sustainable cookie. Ethical aspects such as greater attention to workers and animal welfare are not effective. In the same way, the other dimensions related to sustainability concerns are not statistically significant in influencing the delta.

**Table 6:** SUR parameter estimates (N = 150) with *p*-values in parentheses.

	Model 1		Model 2 *(With participants characteristics)*	
Parameter	Δ Round 1	Δ Round 2	Δ Round 1	Δ Round 2
Trust	0.011 (0.046) **	0.015 (0.022) **	0.036 (0.009) ***	0.048 (0.003) ***
ΔExpectations	0.087 (0.000) ***	0.211 (0.000) ***	0.091 (0.000) ***	0.215 (0.000) ***
Understanding	−0.004 (0.907)	0.009 (0.813)	−0.001 (0.982)	0.008 (0.830)
Productive process concern	0.009 (0.564)	−0.007 (0.722)	0.022 (0.185)	0.006 (0.746)
Environmental concern	−0.006 (0.699)	−0.007 (0.717)	0.005 (0.784)	0.005 (0.811)
Ethical concern	−0.016 (0.321)	−0.032 (0.091) *	−0.018 (0.286)	−0.048 (0.014) **
Women			−0.026 (0.469)	0.033 (0.422)
Age			−0.002 (0.136)	−0.001 (0.372)
Household size			0.001 (0.944)	0.008 (0.570)
Children			−0.038 (0.315)	−0.059 (0.172)
Education			−0.027 (0.456)	−0.100 (0.016) **
Monthly income			0.086 (0.013) **	0.081 (0.040) **
Occupation			−0.031 (0.494)	−0.120 (0.020) **
Cookie shopping frequency			−0.004 (0.800)	−0.013 (0.465)
Average price cookie package			−0.002 (0.952)	0.032 (0.417)
				
*Chi*^*2*^	34.00 (0.000) ***	105.00 (0.000) ***	48.65 (0.000) ***	139.48 (0.000) ***
*Adjusted R*^*2*^	0.19	0.42	0.26	0.51

Note: ***, **, * represent statistical significance at *p* < 0.01, *p* < 0.05 and *p* < 0.1 respectively.

## 4 Discussion

The halo effect generated by sustainable labelling on food packaging has revealed how consumers are driven mainly by intuitive feelings rather than by an analytical processing of the information on the claim; as for organic, fair trade, and similar eco-friendly features. Improved hedonic evaluations and positive expectations of products with the claim (compared to those without) underlie the heuristic “if it's right, it's good” [25,52].

The current study extends the literature on the halo effect by assessing whether the introduction of a completely new, private claim related to sustainable agriculture has a similar capacity on consumers' expectations, interpretation and purchasing behavior. By comparing a sustainable cookies package with its conventional counterpart, this research increases awareness of the potential misleading effect of this sort of claims and its associated consequences.

The first research hypothesis tested whether the private claim generated a halo effect by promoting unjustified sensory and non-sensory expectations of the product and thus, how the claim itself was interpreted in its actual purchase context. The results confirm that the claim-mediated halo effect can create expectations about intrinsic and extrinsic product attributes that have no real motivation [for other examples see, among others, [17, 25, 27]]. Indeed, after detecting the new claim on the bottom left of the package, individuals perceive the cookies package as more particular and attractive, tasting better as well as being of higher quality. Positive subjective evaluations comparable to the enhanced qualities attributed to products with the renowned organic claim [18]. Similarly, although this private claim was not designed to communicate health information or the health properties of ingredients, the sustainable agriculture claim raises such expectations about the final product by framing it as healthier. This finding is in line with the study by Lotz et al. [25] who through three experiments showed how fair-trade claim created a halo of health, despite not providing any calorie or nutritional information about the product. Such confusion could itself lead to negative downstream consequences, as individuals tend to order more food and consume more of it just because it is considered healthier [8,11,53]. These (heightened) expectations derive from the respondent's personal, emphasized interpretation at the sight of the only discriminating factor between the two packages shown, namely the claim “sustainable agriculture”. A probable explanation stems from the fact that the general term sustainability is often associated with vague and confusing interpretations, as it is an abstract concept that simultaneously includes different aspects. When referring to food products, sustainability claims usually cover only one or a few aspects of sustainability and this can lead to misunderstandings when the claim is not known in advance [47]. In our study, about 50% of respondents, when asked to interpret the meaning of the claim, selected at least one unrelated option, associating the claim with additional or completely different processes than the actual claim message (*e.g.,* packaging recycling) or with nuances that intuitively do not fit the cookies production itself (*e.g*., product not tested on animals). This heterogeneity of answers underlines the importance of the halo effect [54]. Consistent with expectations, the abstract concept communicated by the sustainable claim positively influences individuals' perception, whose interest in ethical and social issues creates inferences associating positive connotations with the claim independently of its actual communication [54].

The second research hypothesis tested whether product buyers were willing to pay a premium for the sustainable versus the conventional packaging of cookies. Similar to the existing literature, the results of our study show that respondents are willing to pay a price premium for the sustainable packaging [[28], [29], [30]] in both rounds.

Finally, to investigate whether the halo effect is a central driver of the observed price premium – the third research hypothesis – two SUR regressions were conducted on the sample. The results show that trust in reported certification and expectations are the main factors explaining monetary preferences (both of which can be interpreted in terms of halo effect). The mere presence of a sustainable claim on the packaging justifies a higher price independently of all other aspects. As shown by the high scores of the ethical, environmental, and social concerns related to sustainability, respondents are emotionally involved in sustainable issues and unconsciously reward the product that engages in this direction. Similarly, respondents are willing to pay a premium price for the sustainable cookies as the sensory and non-sensory expectations generated by the product increase. Therefore, these preferences are not due to the actual intrinsic claim message but to something that the claim is not actually communicating. As a consequence of the heuristic that “if it is sustainable, it must be good” [25] and as a result of the complex interaction between emotion and cognition [55].

The results of the current study suggest the potential misleading power of a private claim generally referring to sustainable agriculture.

Although the study results enrich and support the existing literature on consumer preference for food products with sustainable claims, there are several important limitations to consider. These limits concern both the sample included in the experiment (limited, random convenience sample), and specific shortcomings inherent in this type of study. For instance, an important limitation is related to the social desirability bias, *i.e*.: respondents tend to satisfy social norms rather than reveal their true preferences [56]. Similarly, bounded rationality [57,58] and design issues (as the carryover effect and experimental fatigue) could also impact the external validity of current results. Additionally, the label was not launched on the market at the time of the experiment. Moreover, the present experiment does not allow to detect how many participants have simply mistaken the real meaning of the private sustainability claim due to low care or lack of attention during the sessions. Another limit concerns the product chosen for the research, as WTP could be more easily influenced if applied to cheaper sustainable foods (as in the current study) compared to more expensive products [59]. Furthermore, the use of a well-known brand (selected to mimic a real market scenario) could have influenced respondents in their valuations, in terms of previous positive (or negative) reputation and (high/low) trust in the sustainable efforts of the specific food company/brand. Finally, although the BDM mechanism has proven to be very effective, there are several important shortcomings highlighted in literature. Among others, responses may be impacted by price expectations stemming from the prices distribution [60] and individuals may underbid if their interest in the focal product is not very high [61].

## 5 Conclusions

The present study examined if a specific private, sustainability claim on a food product (cookies) would be interpreted to mean something for which it was not designed to communicate and thus affect individual preferences. An artefactual field experiment revealed that a voluntary sustainability claim served as a heuristic cue and influenced consumers' perceptions of several product benefits that are not consistent with its actual features. Additionally, this research revealed that expectations prompted by the sustainability claim positively affected consumers’ monetary preferences, leading to statistically significant premium prices. Therefore, policy makers should thoroughly recognize the most relevant concerns for final consumers and plan effective means to transfer the proper information to the public. Additionally, aiming to better safeguard consumers from deceptive food claims (which lead to information failure) it could be desirable to further explore the possibilities of enforcing current labelling regulations.

However, the halo effect can therefore mislead the valuation and consequently direct to the unaware purchase of the product, as individuals can be willing to pay more for some features that the good does not actually have. Governments and researchers agree that sustainable claims provide consumers with important information to enable them to make informed choices, nevertheless how such labels should be structured and designed is open to debate due to the actual ability of consumers to understand. Producers and policy makers could implement these results in their efforts to encourage the promotion of sustainable food models but aiming to clearer and more easily understandable claims. As effectively noted by Orquin and Scholderer [62] the distance between guiding and misleading consumers is sometimes quite small. Multi-issue claims can be complex and hard to digest for consumers, particularly if some of the communicated sustainability impacts are not commonly associated with the product. Relevance requires the claim maker to communicate only relevant issues, although even this may be too complex if multiple hotspots have been identified.

One important takeaway for marketers is that a sustainable agriculture claim enhances the overall perception of a food product, also persuading many consumers to pay a price premium. Consequently, manufacturers should complement their range of products with a sustainable option to successfully cater the needs of an important segment of the market.

Future research should investigate the effect of a sustainability claim when competing with other existing food claims (as health or/and nutritional). Additionally, the current study incorporated several expectations (as taste, quality, particularity) whilst many others might be considered in a larger experiment. An important extension of present findings could also explore and compare expectations stemming from different private sustainability claims, applied to a diverse range of food products (as fresh or less processed-foods), monitoring brand awareness and its association with the claim message. Further studies should also try to replicate this research in real grocery shopping settings, taking into consideration relevant environmental and situational factors (as product shelf-positioning, point-of-purchase advertisings and offers, time-constraints). Finally, future studies could explicitly explore individuals’ heterogeneity in beliefs and goals applying a larger and more representative sample.

## Author contribution statement

Gerarda Caso: Performed the experiments; Analyzed and interpreted the data; Wrote the paper.

Emanuele Blasi: Conceived and designed the experiments; Contributed reagents, materials, analysis tools or data; Wrote the paper.

Luigi Cembalo: Conceived and designed the experiments; Analyzed and interpreted the data.

Riccardo Vecchio: Conceived and designed the experiments; Analyzed and interpreted the data; Wrote the paper.

## Funding statement

This research was financed through the 10.13039/100010661Horizon 2020 Framework Programme, European Union H2020, founding by project DIVERFARMING – Grant agreement 728003.

## Data availability statement

Data will be made available on request.

## Declaration of interest's statement

The authors declare no competing interests.

## Figures and Tables

**Table A1:** Mean value, standard deviation, and Cronbach's alpha of applied scales.

			Mean	S.D.
SCALE	Trust in sustainable certifications (3 items) ***α = 0.77***	The sustainability certifications represent a real commitment to environmental protection	4.51	1.30
		The promises of product sustainability certifications are probably true	3.95	1.30
		Most of what sustainability certifications say about products is true	3.91	1.29
	*Average*		4.12	1.07
	*Range: from 1 (= totally disagree) to 7 (= totally agree)*			
	Sustainability concern (14 items) ***α = 0.88***	Starvation and malnutrition in the world population	6.36	1.03
		Environmental damage caused by human use of land and water	6.29	1.30
		The amount of food that is wasted	6.27	1.14
		Deforestation of the rain forest	6.17	1.33
		The use of child labour in food production	6.15	1.34
		The use of pesticides used in food production	6.13	1.46
		Poor treatment of animals in food production	5.95	1.47
		Packaging that is not recyclable	5.88	1.48
		Carbon emissions caused by food production	5.85	1.36
		Poor working conditions and wages for food producers	5.69	1.48
		Using too much of the world's natural resources for food production	5.50	1.59
		The amount of packaging used on products	5.41	1.62
		The amount of energy used when transporting food products	4.98	1.61
		The amount of energy used when cooking food products	4.63	1.74
	*Average Range: 1 (= slightly worried) to 7 (= extremely worried)*		5.80	0.89

**Table A2:** Output of Principal-component factors with orthogonal rotation of the 14 items of the Sustainability concern scale.

Items	Factor 1	Factor 2	Factor 3
	*Productive_process*	*Environmental_aspects*	*Ethical_aspects*
	C = 0.24	C = 0.42	C = 0.60
The use of child labour in food production	0.056	0.239	**0.780**
Deforestation of the rain forest	0.116	**0.789**	0.270
Starvation and malnutrition in the world population	0.125	0.308	**0.673**
The use of pesticides used in food production	0.278	**0.634**	0.368
Poor treatment of animals in food production	0.084	0.248	**0.773**
Environmental damage caused by human use (..)	0.256	**0.710**	0.130
The amount of food that is wasted	0.122	**0.577**	0.085
Using too much of the world's natural resources (..)	**0.581**	0.261	0.115
Poor working conditions and wages for producers	0.478	−0.126	**0.615**
Packaging that is not recyclable	**0.565**	0.442	0.186
The amount of packaging used on products	**0.691**	0.347	0.124
Carbon emissions caused by food production	**0.702**	0.071	0.325
The amount of energy used when transporting food	**0.817**	0.141	0.052
The amount of energy used when cooking food	**0.801**	0.126	0.048

Note: The values represent the correlation between the items and generated factors (expressed by factor loading). C represents the cumulative variance explained by the corresponding factor.

**Table A3:** Characteristics associated to the sustainable claim.

	Freq.	%
**Correct answers**		
Promoting sustainable agriculture to help farmers, while protecting the local environment	117	78.00
Using land and water as efficiently as possible to avoid environmental damage	91	60.67
Minimising (soil) contamination when producing food	70	46.67
Minimising chemical emissions when producing goods	47	31.33
Improved conditions for and protection of animals	30	20.00
Ensuring better prices, decent working conditions and good terms for producers	25	16.67
***Incorrect answers***		
Improve packaging and recycling options	34	22.67
Animals are reared outdoors to free range standards	26	17.33
Reducing the amount of packaging used	21	14.00
Ensuring that no child labour is used in the production process	20	13.33
Working to achieve lower prices for consumers	17	11.33
Supporting the production of more local/regional goods	16	10.67
Ensuring that the food produced is distributed in a fair way	15	10.00
Protecting wildlife in the rain forest	13	8.67
Products have not been tested on animals	10	6.67
Don't know	8	5.33

Note: Number and percentage of respondents selecting the statement as describing the claim.

## Appendix A Supplementary data

Survey Cookie_word_V2 Survey Cookie_word_V2

## Footnotes

1 The average cookie package price was retrieved – at the time of the study - from the Italian Department of Economic Development monthly data collections of consumer goods and services called Osserva prezzi (Price Observer). Available online at: https://osservaprezzi.mise.gov.it/.
