# Supplementary material for: This cookie will save the planet! The effect of a private sustainability claim on consumers’ expectations
Source: Heliyon. 2023 Mar 5;9(3):e14206. doi: 10.1016/j.heliyon.2023.e14206 (PMC10023922; doi:10.1016/j.heliyon.2023.e14206)
Supplement: Survey Cookie_word_V2 [file mmc1.docx]

## Full survey (translated from Italian)

[ Screen 1 ]

Enter the ID Code

[ Screen 2 ]

*Preference survey.*

We thank you for agreeing to participate in this survey. The investigation is not for commercial or promotional purposes. The questionnaire is anonymous, the information complies with the privacy law (Decree N.101/2018) and the data collected will not be used for commercial or promotional purposes. Please follow the directions shown in the next screens, complete all the answers and then press the "Send" button.

[ Screen 3 ]

*Food shopping.*

Below you will find some statements, for each we ask you to express your personal opinion. How often do you buy cookies?

- Never
- Rarely
- Sometimes
- Often
- Very often

How much did you pay for the last package of cookies you bought?

- Less than € 1.50
- Between € 1.50 and € 2.00
- Between € 2.00 and € 2.50
- Between € 2.50 and € 3.00
- Over € 3.00

[ Screen 4 ]

*Round 1*


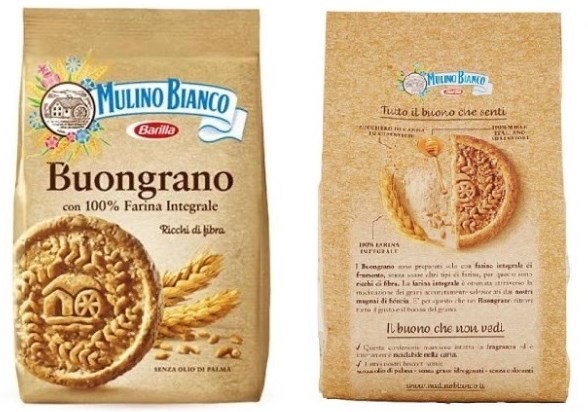

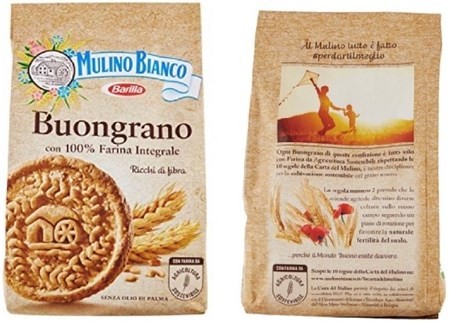


| How much are you willing to pay for the product? | How much are you willing to pay for the product? |
| --- | --- |

[ Screen 5 ]

*Understanding Claim.*

Select all options that you feel valid.

According to you the claim of this product…

- Promoting sustainable agriculture to help farmers, while protecting the local environment
- Improve packaging and recycling options
- Using land and water as efficiently as possible to avoid environmental damage
- Animals are reared outdoors to free range standards
- Minimising (soil) contamination when producing food
- Ensuring better prices, decent working conditions and good terms for producers
- Minimising chemical emissions when producing goods
- Don’t know
- Reducing the amount of packaging used
- Improved conditions for and protection of animals
- Ensuring that no child labour is used in the production process
- Products have not been tested on animals
- Working to achieve lower prices for consumers
- Ensuring that the food produced is distributed in a fair way
- Supporting the production of more local/regional goods
- Protecting wildlife in the rain forest

[ Screen 6 ]

*Below we give you a very short text that we ask you to read carefully*

It is thanks to nature, organized in eco-systems, that we can drink, care, eat, warm up and relax looking at a beautiful landscape or walking in a park. For more than 250,000 years, ecosystems and their interactions have guaranteed us the daily and free supply of goods and services, so-called ecosystem services, which influence our well-being in a more or less direct way.. Globally these services have been classified into 4 types:.

- life support services (nutrient cycle, photosynthesis, soil formation);.
- supply services (food, drinking water, active ingredients of medicines, fuels and materials);.
- regulating services (climate and tides, water purification, pollination and biodiversity);
- cultural services (mental health, aesthetic, spiritual, educational and recreational values).

The cookies Buongrano made with flour from sustainable agriculture, safeguard ecosystems favoring the normal fertility of the soil.

[ Screen 7 ]

*Round 2*


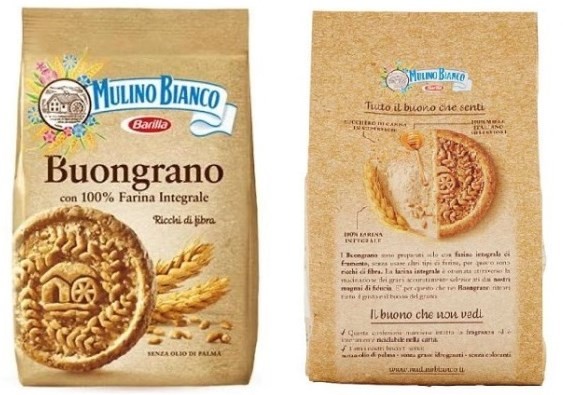

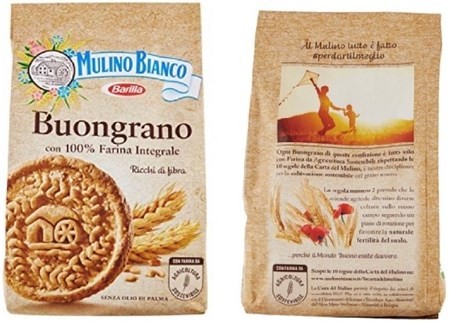


| How much are you willing to pay for the product? | How much are you willing to pay for the product? |
| --- | --- |

[ Screen 8 ]

*Expectations*


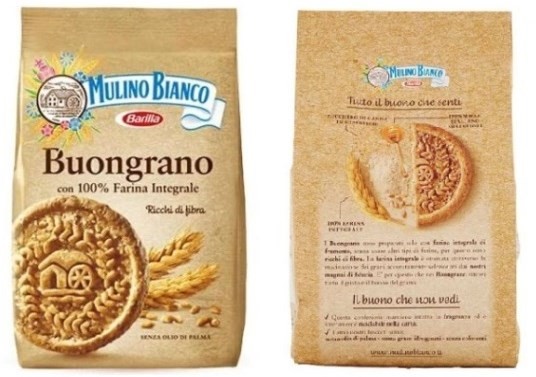


We ask you to express your opinion about each of these statements (Scale from 7=much better to 1=much worst).

|  | 1 | 2 | 3 | 4 | 5 | 6 | 7 |
| --- | --- | --- | --- | --- | --- | --- | --- |
|  | Much worse |  |  | Equal |  |  | Much better |
| I expect the Quality of this product compared to other similar products to be. | O | O | O | O | O | O | O |
| I expect the Particularity of this product compared to other similar products to be. | O | O | O | O | O | O | O |
| I expect the Attractiveness of this product compared to other similar products to be. | O | O | O | O | O | O | O |
| I expect the Taste of this product compared to other similar products to be. | O | O | O | O | O | O | O |
| I expect the Nutritional properties of this product compared to other similar products to be. | O | O | O | O | O | O | O |
| I expect the Safety of this product compared to other similar products to be. | O | O | O | O | O | O | O |
| I expect the Healthiness of this product compared to other similar products to be. | O | O | O | O | O | O | O |

[ Screen 9 ]

*Expectations*


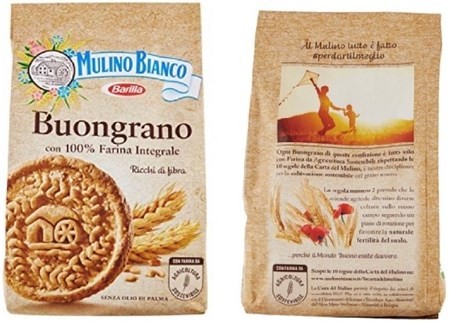


We ask you to express your opinion about each of these statements (Scale from 7=much better to 1=much worst).

|  | 1 | 2 | 3 | 4 | 5 | 6 | 7 |
| --- | --- | --- | --- | --- | --- | --- | --- |
|  | Much worse |  |  | Equal |  |  | Much better |
| I expect the Quality of this product compared to other similar products to be. | O | O | O | O | O | O | O |
| I expect the Particularity of this product compared to other similar products to be. | O | O | O | O | O | O | O |
| I expect the Attractiveness of this product compared to other similar products to be. | O | O | O | O | O | O | O |
| I expect the Taste of this product compared to other similar products to be. | O | O | O | O | O | O | O |
| I expect the Nutritional properties of this product compared to other similar products to be. | O | O | O | O | O | O | O |
| I expect the Safety of this product compared to other similar products to be. | O | O | O | O | O | O | O |
| I expect the Healthiness of this product compared to other similar products to be. | O | O | O | O | O | O | O |

[ Screen 10 ]

*Trust in sustainable certifications*

Below you will find some statements, for each we ask you to declare your degree of agreement or disagreement, with a score from 1= totally disagree to 6=totally agree.

|  | 1 | 2 | 3 | 4 | 5 | 6 |
| --- | --- | --- | --- | --- | --- | --- |
|  | Totally disagree |  |  |  |  | Totally agree |
| The sustainability certifications represent a real commitment to environmental protection | O | O | O | O | O | O |
| The promises of product sustainability certifications are probably true | O | O | O | O | O | O |
| Most of what sustainability certifications say about products is true | O | O | O | O | O | O |

[ Screen 11]

*Concern about sustainability issues in food production.*

Below we ask you to express your level of concern about some issues, 1 = slightly worried, 7 = extremely worried.

|  | 1 | 2 | 3 | 4 | 5 | 6 | 7 |
| --- | --- | --- | --- | --- | --- | --- | --- |
|  | Slightly worried |  |  |  |  |  | Extremely worried |
| Starvation and malnutrition in the world population | O | O | O | O | O | O | O |
| Environmental damage caused by human use of land and water | O | O | O | O | O | O | O |
| The amount of food that is wasted | O | O | O | O | O | O | O |
| Deforestation of the rain forest | O | O | O | O | O | O | O |
| The use of child labour in food production | O | O | O | O | O | O | O |
| The use of pesticides used in food production | O | O | O | O | O | O | O |
| Poor treatment of animals in food production | O | O | O | O | O | O | O |
| Packaging that is not recyclable | O | O | O | O | O | O | O |
| Carbon emissions caused by food production | O | O | O | O | O | O | O |
| Poor working conditions and wages for food producers | O | O | O | O | O | O | O |
| Using too much of the world’s natural resources for food production | O | O | O | O | O | O | O |
| The amount of packaging used on products | O | O | O | O | O | O | O |
| The amount of energy used when transporting food products | O | O | O | O | O | O | O |
| The amount of energy used when cooking food products | O | O | O | O | O | O | O |

[ Screen 12 ]

*Socio-demographic information* Please answer these last questions Area of residence

Age

Gender

Number of family members

- 1 component
- 2 components
- 3 components
- 4 components
- 5 components
- More than 5 components

Children in the household

- Yes
- No

Education

- Elementary School
- Secondary school
- High school diploma
- Bachelors degree
- Master / PhD

Household monthly income

- Low
- Medium
- High

Occupation

- Entrepreneur
- Employee
- Freelancer
- Housewife
- Retiree
- Teacher
- Unemployed
